# Small-Scale Morphological Features on a Solid Surface Processed by High-Pressure Abrasive Water Jet

**DOI:** 10.3390/ma6083514

**Published:** 2013-08-14

**Authors:** Can Kang, Haixia Liu

**Affiliations:** 1 School of Energy and Power Engineering, Jiangsu University, Zhenjiang 212013, China; E-Mail: kangx603@umn.edu; 2 School of Material Science and Engineering, Jiangsu University, Zhenjiang 212013, China

**Keywords:** high-pressure water jet, abrasive particle, turbulent flow, jet-cutting experiment, morphological feature, surface roughness

## Abstract

Being subjected to a high-pressure abrasive water jet, solid samples will experience an essential variation of both internal stress and physical characteristics, which is closely associated with the kinetic energy attached to the abrasive particles involved in the jet stream. Here, experiments were performed, with particular emphasis being placed on the kinetic energy attenuation and turbulent features in the jet stream. At jet pressure of 260 MPa, mean velocity and root-mean-square (RMS) velocity on two jet-stream sections were acquired by utilizing the phase Doppler anemometry (PDA) technique. A jet-cutting experiment was then carried out with Al-Mg alloy samples being cut by an abrasive water jet. Morphological features and roughness on the cut surface were quantitatively examined through scanning electron microscopy (SEM) and optical profiling techniques. The results indicate that the high-pressure water jet is characterized by remarkably high mean flow velocities and distinct velocity fluctuations. Those irregular pits and grooves on the cut surfaces indicate both the energy attenuation and the development of radial velocity components in the jet stream. When the sample is positioned with different distances from the nozzle outlet, the obtained quantitative surface roughness varies accordingly. A descriptive model highlighting the behaviors of abrasive particles in jet-cutting process is established in light of the experimental results and correlation analysis.

## 1. Introduction

When water is pressurized by a high-pressure reciprocal pump and then guided through an orifice of 0.25–0.40 mm in diameter, a tiny, high-velocity jet stream will be attained. An abrasive water jet (AWJ) is produced, provided that abrasives, such as quartz sands and silicon carbide particles, are mixed into the water before it is ejected from the nozzle. With high-pressure water-jet technology, a myriad of engineering applications, such as cutting, drilling and milling have been achieved. High-pressure water-jet technology has attracted lots of attention from both fluid dynamics and material engineering fields, especially in recent years [1,2]. In addition, a high-pressure water jet system is uniquely advantageous for its capability of avoiding a thermal distortion effect with respect to the processed object. 

There are two kinds of commonly used water jets, namely, the water jet and abrasive water jet. The performance of both the two jets is unanimously determined by the flow features inside the jet stream [3]. However, experimental studies in the fluid dynamics aspects of a high-pressure water jet have rarely been reported, which is partially due to the complexity in the measurement of such a tiny and high-velocity jet stream (the diameter of the jet stream near the nozzle’s outlet section is approximately 1.0 mm, and the local average flow velocity may exceed 800 m/s). In the meantime, lots of efforts are being devoted to the investigation of the influence of operating parameters, such as jet pressure, stand-off distance (SOD), traverse speed of nozzle and the nozzle’s deflection angle [4,5,6]. It should also be noted that variation of the sample’s material or dimensions will necessitate more detailed experiments and comparative analysis before an optimal combination of those operating parameters is attained [7,8]. 

Regarding the most immediate impact on a target surface, both longitudinal and radial jet velocity components are critical, which reasonably explains the importance of many relevant works. Computational fluid dynamics (CFD) technique allows for the prediction of turbulent parameter distributions in the jet stream and provides an effective tool for the study of high-pressure water jets [9,10]. However, in light of the published numerical studies of high-pressure water jets, those aspects, such as feasibility of multiphase turbulent models, jet interface stability and the droplet’s dynamic behavior, have not been soundly treated [11,12]. Moreover, simultaneous computation of both flow field and solid deformation is greatly anticipated, although the complexity level of the numerical work will be evidently elevated, thereby. In view of this, the Bernoulli equation or empirical formula is usually relied upon to yield an average jet velocity, which is often treated as the initial condition in the analysis of material stress or potential fracture of material. The relation built empirically between *v*, the jet velocity at the nozzle outlet, and *p*, the jet pressure, can be given by:

(1) $$v=44.7\sqrt{p}$$

where the units of *v* and *p* are m/s and MPa, respectively.

The essential relationship between jet flow and the solid surface processed by the jet flow is crucial for such an interdisciplinary subject, which serves as a primary incentive to the present study. As an important prerequisite, the non-intrusive flow measurement of a water jet at jet pressure of 260 MPa is performed. The measurement is valuable in terms of quantitatively determining the impact effect on sample surface and correlating highly turbulent jet flow with surface morphology. Distributions of principal turbulent flow parameters, such as mean velocity and root-mean-square velocity at the jet sections near nozzle’s outlet section, are of great importance. Phase Doppler anemometry is utilized to acquire these turbulent flow parameters. Then, abrasives are added to the water jet, and samples of Al-Mg alloy are processed by the liquid-solid two-phase jet stream. Consequential surface morphology and roughness are treated by utilizing a scanning electron microscope and an optical profiling system. The morphological features are expected to be correlated with inherent fluid dynamics characteristics in the high-velocity jet stream. On the basis of the explanation of the momentary interaction between abrasive particles and cut surface, a descriptive model highlighting the behaviors of abrasive particles in the course of a jet-cutting operation is established. 

## 2. Experimental Set-Up 

### 2.1. High Pressure Water Jet System 

An ultra-high pressure system manufactured by Dardi International Corporation of China served as the source of high pressure in the experiment. With a single intensifier, a maximum jet pressure of 380 MPa could be reached. The primary operating parameters of the system are listed in Table 1. The nozzle was fixed at a supporting frame, whose traverse and vertical displacement were accurately manipulated through a digital control panel. 

**Table 1:** Primary operating parameters of the ultra-high pressure system.

Parameter	Description
Pressurization ratio	20:1
Power	30 kW (50 Hz)
Maximum discharge pressure	380 MPa
Maximum flow rate of water	3.7 L/min

### 2.2. Phase Doppler Anemometry Technique

As a non-intrusive measurement technique, phase Doppler anemometry has been widely recognized as an accurate and effective instrument for measuring flow velocity and droplet size. According to the light-wave Doppler effect in Einstein’s special theory of relativity, when an object scatters lights, consequent Doppler frequency shift could be associated with the traveling speed of the object. Velocity component, *v_i_*, of the object can be given by:

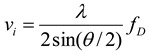
(2)
Where *λ* is the wavelength of incident light, *f_D_* is Doppler frequency and *θ* is the angle between the two incident laser beams. Droplet diameter, *D*, can be obtained by the following function:

*D* = *f*(*ϕ*,*n*,*λ*,*β*) (3)

Where *ϕ* is the phase difference between Doppler bursts, *n* is the refractive index of the scattering medium and *β* is a constant relevant to the scattering mode. There is a linear relationship between droplet diameter and Doppler signal phase.

**Figure 1:**
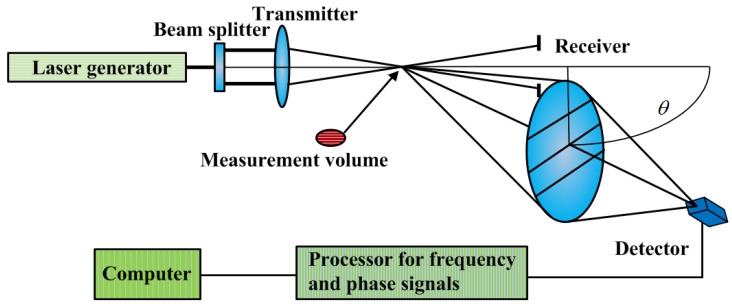
Schematic diagram of phase Doppler anemometry system and jet flow measurement.

In a PDA system, after passing through the fiber driver, the single-wavelength laser generated by a light source is divided into a green laser beam and a blue laser beam. Through a transmitter probe, two vertically separated green laser beams are eradiated for the measurement of axial velocity and droplet diameter. Two horizontally separated blue laser beams are eradiated for the measurement of radial velocity. When it comes to the measurement of jet flow, the laser beams will be focused on one point in the jet flow field, and an ellipsoidal measurement volume is produced thereby (see Figure 1.).

In view of the state-of-the-art non-intrusive measurement, it is quite difficult to perform an accurate PDA measurement of a high-pressure abrasive water jet flow when jet pressure exceeds 160 MPa. The problems arising by partial or entire overlaps among abrasive particles in the tiny jet stream will give rise to dubious results of abrasive particle’s velocity. Furthermore, it is even harder to extract the transient sectional distribution of abrasive particles of various shapes. In that case, we assume that at the same spatial position, mean abrasive particle’s velocity is identical with water’s velocity in terms of both magnitude and direction. Since the studied sections here are in close vicinity to the nozzle outlet, the assumption can be deemed as feasible. Based on this assumption, PDA measurement is carried out with working fluid of water. Those minute impurity droplets of about 20 μm in diameter contained in the water are utilized as seeding droplets.

**Figure 2:**
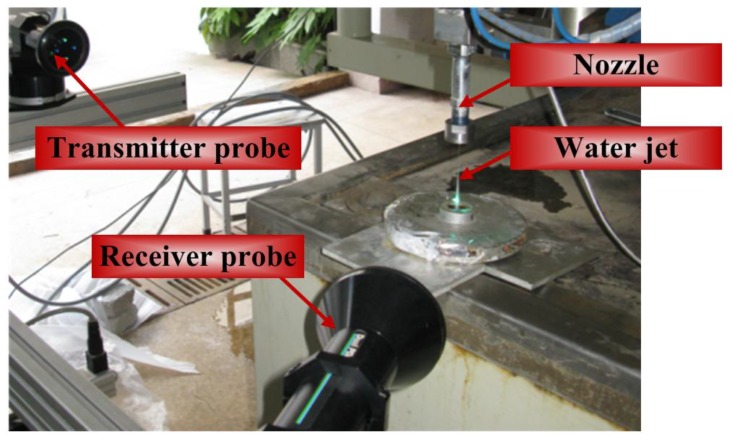
Configuration of the experimental components.

The configuration of a made-in-house nozzle, the transmitter and receiver probes of a Dantec PDA system are presented in Figure 2. The forward scattering model of refraction is adopted in light of the medium properties. The transmitter and receiver probes in Figure 2 are connected to a computer-controlled traverse system. A green laser with its wavelength of 514.5 nm and a blue laser with its wavelength of 488.0 nm are radiated. The dimensions of the measurement volume produced by the green laser are 0.243 mm × 0.243 mm × 6.389 mm, while the dimensions of the measurement volume produced by the blue laser are 0.230 mm × 0.230 mm × 6.060 mm.

Since the diameter of the nozzle’s outlet section, *d*, is 0.8 mm, the measurement volumes’ dimensions can fulfill the requirements of the measurement. Generally speaking, desirable impact by a jet stream results exclusively from the jet stream segment which is within the distance of 10*d* from the nozzle’s outlet section. Beyond that distance, the jet stream gets expanded distinctly, and the kinetic energy contained in the jet stream attenuates considerably. In this study, at the constant jet pressure of 260 MPa, the two sections at respective stand-off distances of 3.0 mm (3.75*d*) and 7.0 mm (8.75*d*) are investigated.

Regarding the fluid dynamics aspects of high-pressure abrasive water jet, there are diversified viewpoints on multiphase flow physics and the interaction between liquid and solid phases. The assorted situations in the jet flow can be traced back to the mixing chamber, where the flow becomes complicated immediately after the mixing of pure water with abrasive particles [13]. In this experiment, the slip between abrasive particles and water in the jet stream segment concerned with is not taken into consideration, because the tiny and concentrated jet stream plays a deterministic role in restricting the growth of such an inter-phase slip effect. As with those downstream positions sufficiently far from the nozzle’s outlet section, the slip effect unquestionably deserves a close inspection.

### 2.3. Validation of Sampling Process

**Figure 3:**
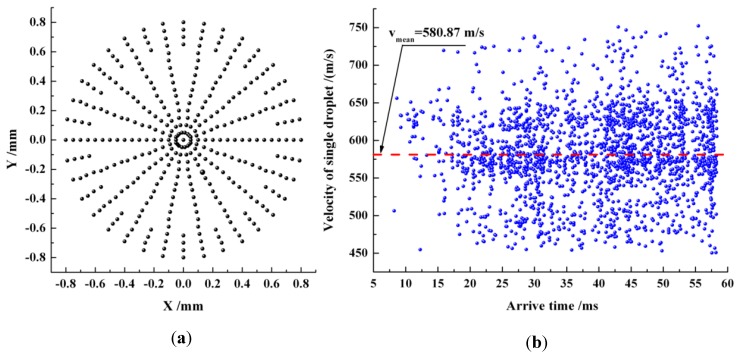
Arrangement and validation of measurement volume. (**a**) Positions of measurement volumes on a studied section; (**b**) distribution of the velocities of those droplets passing through the measurement volume deployed at the center of *Z* = 3.0 mm section within 57 ms.

With reference to some monitored section of the jet stream, those points shown in Figure 3a are anticipated to be experienced sequentially by the optical focus. When the jet flow got fully developed, those droplets successively passing through the measurement volume located at the center of the *Z* = 3.0 mm section were recorded within in a time span of 57 ms to justify the velocity measurement. Here, the jet stream propagates in the *+Z* direction, and the origin of the *Z*-axis is situated at the center of the nozzle’s outlet section. There are roughly 2600 captured droplets, and their velocities and diameters are acquired simultaneously. The process of acquisition of droplet velocities is explained in Figure 3b, where the arrival time denotes the moment when droplet arrives at the measurement volume.

Velocity magnitudes in Figure 3b cover a wide range, and the discrepancy between the maximum and minimum velocities exceeds 280 m/s. The distribution of those droplets with peak velocities is relatively sparse, and a large majority of droplets gather near the dash line indicating mean velocity, with their velocities ranging from 500 to 650 m/s. The arithmetic mean velocity of 580.87 m/s is obtained by using a statistical algorithm. Such a sampling process was repeated three times in the same measurement volume. The maximum relative deviation among the three mean velocity magnitudes obtained, thereby, was proven to be lower than 5%. 

## 3. Discussion of Jet Flow Characteristics

### 3.1. Distribution of Mean Velocity

**Figure 4:**
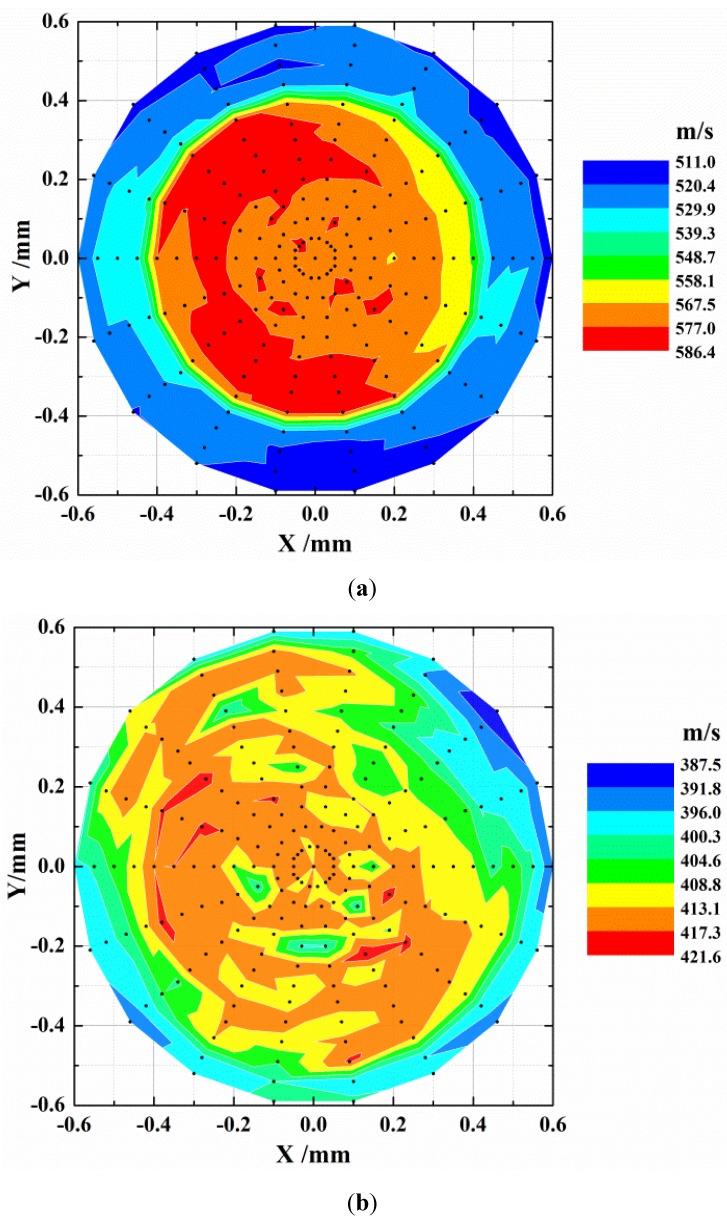
Distributions of mean velocity at (**a**) *Z* = 3.0 mm and (**b**) *Z* = 7.0 mm.

The distribution of mean velocity at the section of *Z* = 3.0 mm is shown in Figure 4. The most salient feature of the distribution lies in the circular protrusion zone of high velocity at the central part of the section. The diameter of that zone is about 0.8 mm, which is equivalent to the diameter of the nozzle’s outlet section. In contrast, those mean velocity magnitudes in the neighboring zone surrounding the central zone are relatively low. There is a seemingly distinct velocity gradient at the interface between the two kinds of zones. Additionally, the distribution of mean velocity in the protruded high-velocity zone is not uniform with respect to such a small velocity scale, which cannot be attained with commonly used numerical methods. Moreover, it can be reasonably concluded from Figure 4a that the strong cutting force of concentrated jet stream originates from such a high level of kinetic energy.

By virtue of the same manner of expression as that in Figure 4a, relatively diffused velocity distribution at *Z* = 7.0 mm section is shown in Figure 4b. Here, overall velocity magnitude declines considerably with the increase of the longitudinal distance from the nozzle’s outlet section. There is still a discernible high-velocity central zone, to which a non-uniform velocity distribution is attached, as well. It is noticeable that the diameter of the high-velocity zone in Figure 4b is enlarged compared to its counterpart in Figure 4a, which furnishes a further explanation of the diffusion and attenuation of fluid’s kinetic energy. Simultaneously, with the lateral expansion of jet stream, the cutting ability of jet stream is weakened to some extent and radial velocity component tends to be noticeable. According to preceding assumptions, the mean velocity of water obtained here will be ultimately transferred to abrasive particles with the necessary spatial correspondence being taken into account. 

### 3.2. Root-Mean-Square Velocity

**Figure 5:**
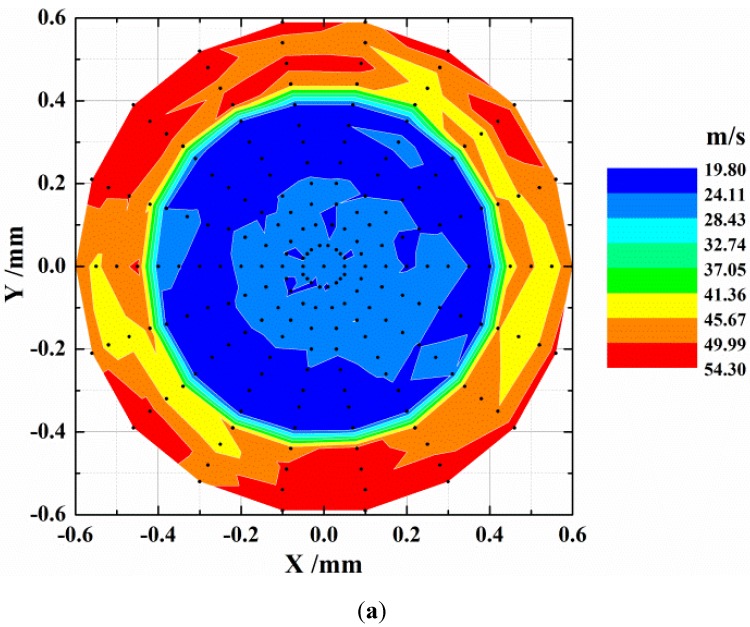

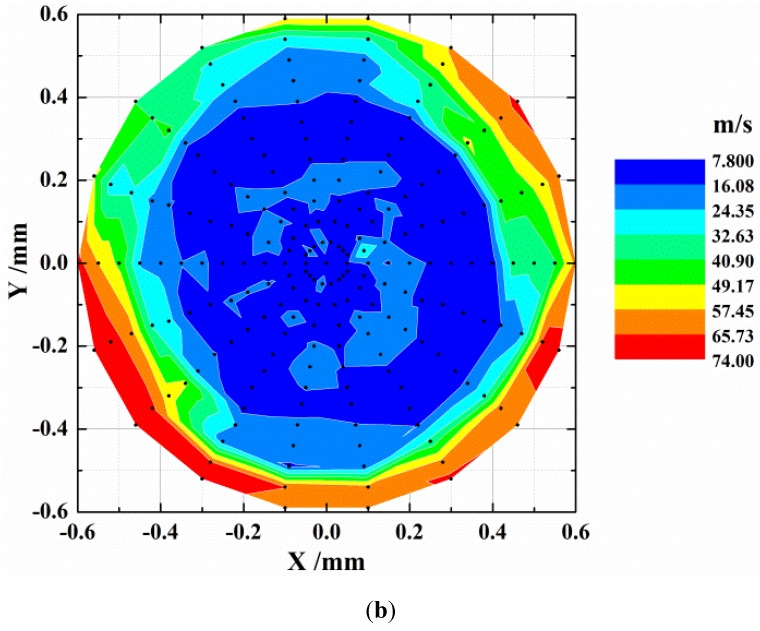
Distributions of root-mean-square velocity at (**a**) Z = 3.0 mm; and (**b**) Z = 7.0 mm.

The quantity of velocity can generally be decomposed into two components, namely, mean velocity and fluctuating velocity. The fluctuating velocity has a strong effect on the momentum transfer between the jet flow and the droplets or particles involved [14]. Unfortunately, such an effect has not been expressed in an accessible manner until now, which is predominantly ascribed to limited knowledge of the very nature of turbulence. In most cases, root-mean-square velocity is introduced to express the velocity fluctuation at certain measurement volume and can be given by:

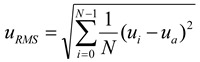
(4)
where *N* is the number of velocity data, *u_i_* indicates droplet velocity and *u_a_* is the mean velocity with respect to the entire measurement volume. Distributions of RMS velocities at sections of *Z* = 3.0 mm and *Z* = 7.0 mm are shown in Figure 5a,b, respectively.

Root-mean-square velocity is closely linked to the distribution of mean velocity, as is testified by the comparison between Figure 4 and Figure 5. In the central parts of the two sections, an RMS velocity valley is unanimously found, albeit with different profiles. In Figure 5a, variation of RMS velocity in the central part of the section is relatively smooth compared with that in Figure 5b. Due to a higher velocity magnitude and a more uniform RMS velocity distribution, the jet flow at the section of *Z* = 3.0 mm is capable of producing a stronger and more consistent impact effect. With regard to the PDA measurement, there are several other aspects, such as jet stability and liquid-jet breakup, that may influence the jet’s impact on a target surface [15,16], which is beyond the scope of this study. 

## 4. Abrasive Water Jet Experiment

With respect to jet-cutting experiment, numerous studies have been undertaken to illustrate the relationships between various operational parameters and the obtained results, which also reflects the diversity aroused by diversified materials and combinations of those operational parameters [17,18]. The jet-cutting experiment here is specially devised to construct a correlation between the fluid dynamics aspects and the material’s response to the jet flow.

### 4.1. Liquid-Solid Two-Phase Jet Nozzle

**Figure 6:**
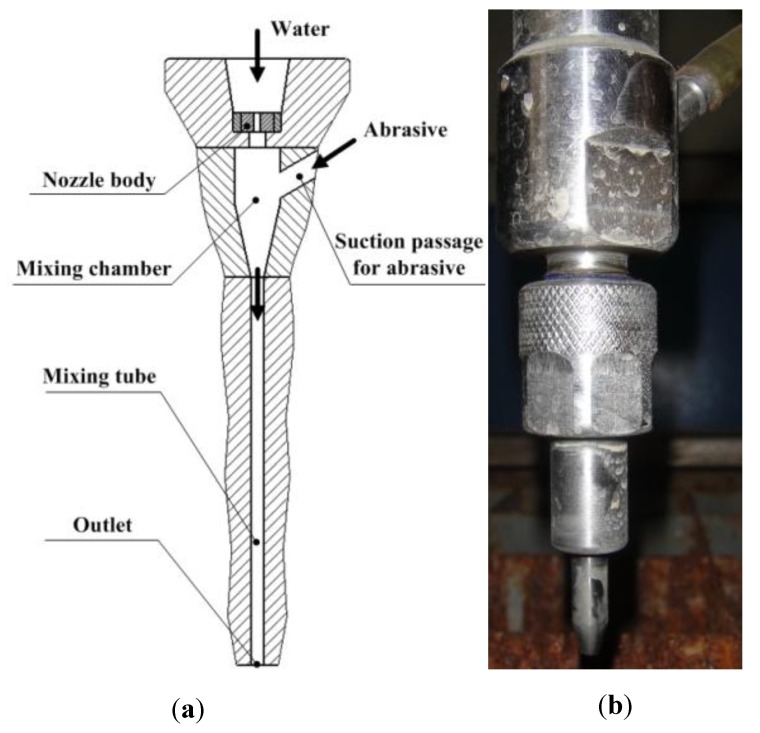
Liquid-solid two-phase jet nozzle. (**a**) Schematic view; (**b**) image of the nozzle.

In order to entrain abrasive particles into the water flow by a pressure-difference effect to produce an abrasive water jet, the liquid-solid two-phase nozzle presented in Figure 6 is frequently used in jet-cutting experiments. Local static pressure in the mixing chamber is rather low, due to large velocity magnitudes. Therefore, the pressure difference will drive the abrasive particles’ movement toward the mixing chamber, and a mixture of water and abrasive particles will be acquired, thereby, in the mixing chamber. There is a long straight mixing tube before the nozzle’s outlet section, and liquid-solid two-phase flow will get fully developed before it is ejected from the nozzle. The diameter of the nozzle’s outlet section was 0.8 mm, which was identical with that adopted in the flow measurement.

### 4.2. Abrasive and Sample

Quartz sands with equivalent diameter of 0.177 mm served as abrasive particles in the experiment. The transverse speed of the nozzle was 200 mm/min. At a jet pressure of 260 MPa, stand-off distances of 3.0 mm and 7.0 mm were separately adopted to acquire comparable results. Jet direction was set to be perpendicular to the target surface. The flow rate of abrasive particles was 520 g/min. All cubic samples of Al-Mg 5A02 alloy had the identical dimensions of 30.0 mm × 8.0 mm × 8.0 mm, and the square section was positioned to be parallel to the jet direction. In addition, the samples were reasonably assumed to be identical in terms of their physical properties.

### 4.3. Observation and Analysis of Cut Surface 

**Figure 7:**
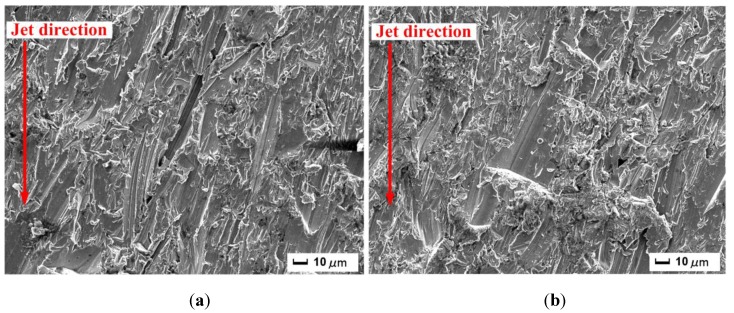
Surface morphological features on cut sections: (**a**) *Z* = 3.0 mm; (**b**) *Z* = 7.0 mm.

Two samples were processed by abrasive water jet when the target surfaces were positioned at *Z* = 3.0 mm and 7.0 mm, respectively. Here, the surfaces are visualized through a scanning electron microscope, which has been employed as an effective method in describing the small-scale features on cut surface at varying magnification factors [19]. From those images acquired by utilizing such a technique, two typical images representing the results at different standoff distances respectively are displayed in Figure 7. In both Figure 7a,b, apparent traces created by abrasive particles can be found, and distributions of traces are relatively uniform in Figure 7a. In Figure 7b, there is a wide groove in the middle of the image that terminates its progress with a truncation end, which is one of the most distinct features on the surface being exposed to abrasive water jet. The amount of kinetic energy is not sufficient for the abrasive particles to cut through the material, and the abrasive particles may possibly deviate from their original motion directions or directly embed themselves in the sample. From another point of view, lateral expansion of jet stream prompts the development of radial velocity component in the jet stream. Consequently, the radial velocity component enhances to some degree the possibility of the embedment of abrasive particles. Such a phenomenon has been investigated in other literature as well [20]. It should also be noted that physical properties of abrasive particles cannot be neglected in this connection. 

### 4.4. Surface Roughness Analysis

Surface roughness is a significantly valuable parameter in engineering applications and often relied upon to evaluate the surface quality of processed solid work piece [21]. Apparently, those surface roughness testers commonly used in engineering fields cannot fulfill the current requirements, because of the coexistence of regularity and irregularity on the cut surface. Here, the surface roughness is measured using the Wyko NT1100 optical profiling system. The white-light vertical scanning interferometry (VSI) is adopted to detect the vertical height values on the cut surfaces. The predefined vertical resolution is 5 nm. Based on a huge amount of sparse data indicating the height values on the surfaces investigated, two representative images are constructed to describe the morphological features on the two surfaces obtained at stand-off distances of 3.0 mm and 7.0 mm, respectively. 

**Figure 8:**
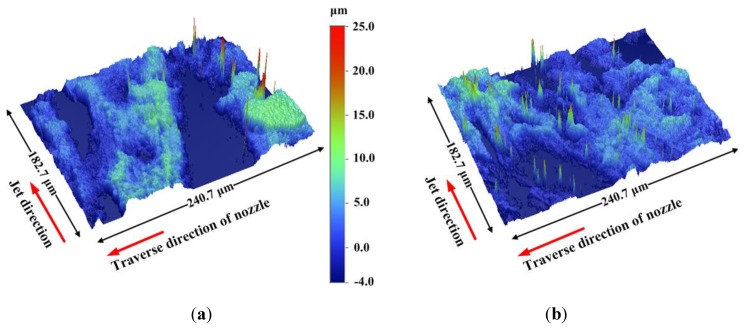
Three-dimensional surface morphological features: (**a**) *Z* = 3.0 mm; (**b**) *Z* = 7.0 mm.

As shown in Figure 8, the irregularity of processed surfaces lies primarily in the high and low sub-zones produced by the abrasive particles. Repeated actions on the same position by high-speed abrasives are evidenced by the wide groove in Figure 8a, since the maximum width of the groove has obviously exceeded the equivalent diameter of the abrasive particles used. In both Figure 8a,b, assorted pits are witnessed. In view of the interaction between abrasive particles and target sample, the originally uniform motion direction of the abrasive particles is altered because of twin factors: one is the insufficient kinetic energy carried by the abrasive particles and the other is the rebound effect occurring when abrasive particles hit the sample surfaces. Figure 8b is characterized by more pits and discontinuous grooves. It can be manifested by the morphological features in Figure 8b that surface quality will deteriorate with the distance from the nozzle’s outlet section, and more abrasive particles bear remarkable radial velocity components, along with the lateral expansion of jet stream. 

**Figure 9:**
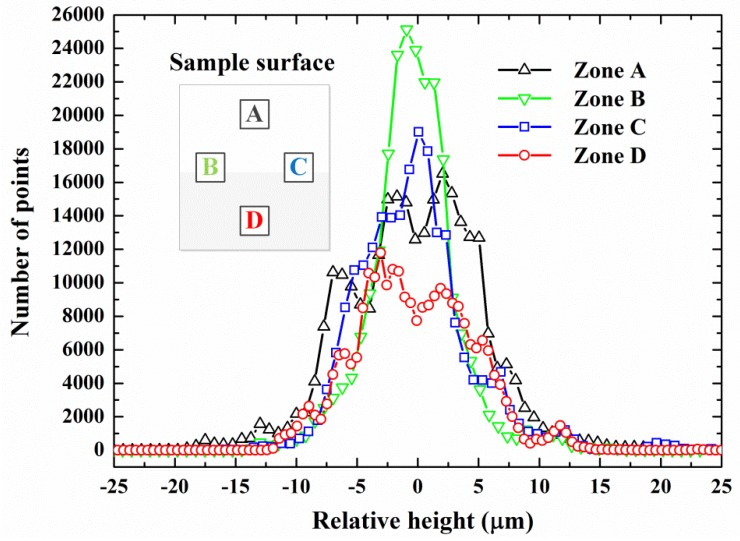
Statistical relation based on the morphological data (for comparison, a base plane where the relative height is predefined to be zero is identically set for the four zones.).

Further analysis is made possible with the morphological data obtained by using the optical profiling system. The data of the four zones on the surface treated at *Z* = 7.0 mm are separately extracted and, then, analyzed statistically. As shown in Figure 9, Zone A is the closest to the nozzle’s outlet section (the distance is 7.5 mm), and Zone D is the farthest from the nozzle (the distance is 10.5 mm). The uniform distance from the nozzle’s outlet section to Zone B and Zone C is 9.0 mm. The total number of points recorded in each of the four zones is identical, and the same horizontal base plane is defined for the comparison of those relative-height values in the four zones. Figure 9 contains the number of points corresponding to various relative-height values. It can be found in Figure 9 that the profile representing the statistical relationship in Zone A deviates clearly from that in Zone D. The relative-height values of most sampling points in Zone A are centralized in the relative-height-value range of −5 μm to 5 μm. As with Zone B and Zone C, the distribution tendency is comparatively similar. Such statistical results provide quantitative and elaborate evidence for the effects of high-speed abrasive particles on target surfaces. Furthermore, the average surface roughness of each zone can be exactly calculated on the basis of the acquired data. 

### 4.5. Descriptive Model of Jet-Cutting Process

**Figure 10:**
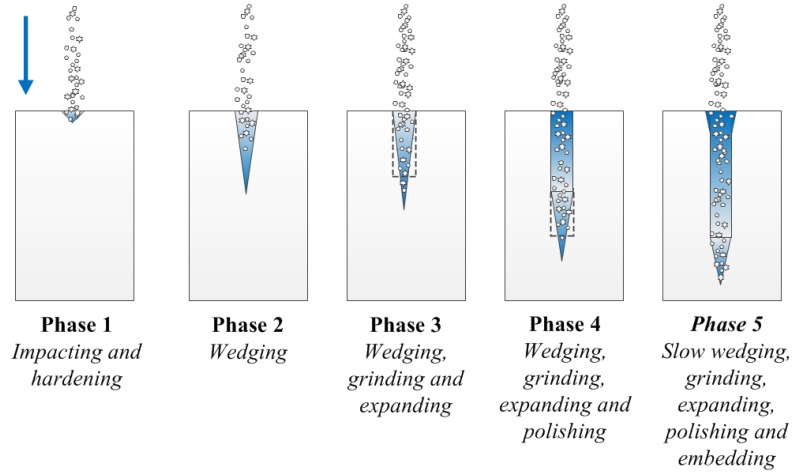
Five phases in the jet-cutting process (the blue kerf is assumed to develop primarily in the longitudinal direction).

With reference to ordinary mechanical cutting, analytical models have been established to illustrate the various effects in the course of the mechanical interaction between cutting tool and work piece [22]. Those models are especially instructive when handling materials with different physical properties. There is no available model to describe the details regarding the momentary phenomena occurring in the jet-cutting process, which is largely due to the considerable discrepancy between the results obtained in theoretical reasoning and engineering applications. From the preceding analysis, we are capable of building a descriptive model to depict various abrasive particles’ behaviors in the vicinity of the cut surface. Schematic explanation of such a model is indicated in Figure 10. 

There are five sequential phases that will be experienced in the jet-cutting process. With the adjustment of operation parameters, such as stand-off distance, jet pressure and traveling speed of nozzle, the duration of each phase may vary accordingly. It should be noted that the time scale in the jet-cutting process is rather small, as can be predicted in light of the magnitude of abrasive particle’s velocity. Any modulation of operation parameters may give rise to assorted surface morphological features, which has been proven in a myriad of experiment studies [18].

Here, the size distribution and volume fraction of abrasive particles are not taken into account to avoid the complex uncertainties provoked thereby [23]. In Figure 10, descriptions are made to reflect the behaviors of abrasive particles and the features of resultant kerf. Certainly, in Phases 2 and 3, the issue of embedment of abrasive particles may also emerge on the cut surface. Because of the continuous supplement of abrasive particles, the embedded abrasive particles may be impacted by the subsequent abrasive particles, and the embedded abrasive particles may even have the risk of being scaled off from the surface to which they are attached.

In some previous numerical studies, the model of material-removing has been underscored. However, those small-scale and transient phenomena relevant cannot be explained with existing measurement and visualization techniques [24,25]. The predictable behaviors enumerated in Phase 5 necessitate lots of experiments before they are ultimately elucidated. With the traveling of the nozzle, the abrasive particles’ influence on target sample is eventually terminated by abrasives’ being trapped in cut surface or leaving with the removed solid material. The contribution of the water in jet stream is not explored in this respect, and one of the most important functions of the water lies in carrying away the fragments of removed material and abrasive particles.

## 5. Conclusions

The abrasive water jet technique is very promising in terms of its unique merits, necessary for many engineering applications. However, several crucial points in both turbulent flow and the interaction between abrasive particles and impacted solid surface are kept partially or completely unrevealed. Such an interdisciplinary subject requires more efforts to be invested to the observation and analysis of those small-scale spatial and temporal phenomena. The primary conclusions that can be drawn from the study are as follows.

(1) At a jet pressure of 260 MPa, water jet velocity is quantitatively measured with non-intrusive PDA technique. At the section of *Z* = 3.0 mm, the maximum mean velocity of water exceeds 560 m/s, which confirms the material processing capability of high-pressure water jet. With the downstream propagation of the jet stream, both jet stream’s lateral expansion and the attenuation of fluid’s kinetic energy are manifested. 

(2) Abrasive particles inherit the attribute of mean velocity from the water jet, which is the prerequisite for the analysis of the interaction between abrasive particles and impacted Al-Mg alloy samples. At different stand-off distances, the resultant cut surfaces are featured by irregularity of different extents. Various grooves and pits on the cut surfaces reflect both the evolution of kinetic energy and radial velocity component on the part of abrasive particles. 

(3) Morphological features become more salient with the increase of longitudinal distance from the nozzle’s outlet section. Quantitative conclusions are achieved through statistical analysis, and the distributions of relative-height values on cut surfaces are associated with detailed abrasive behaviors. The five-phase model describing the abrasive particles’ behaviors in the jet-cutting process is reasonably established in light of the experimental results. Further exploration of this subject incorporating more flow-related factors is anticipated to be carried out based on the conclusions here.
